# KLF transcription factors in bone diseases

**DOI:** 10.1111/jcmm.18278

**Published:** 2024-03-28

**Authors:** Haixia Wang, Juanjuan Han, Gorbachev Dmitrii, Ke Ning, Xin‐an Zhang

**Affiliations:** ^1^ College of Exercise and Health Shenyang Sport University Shenyang Liaoning China; ^2^ Department of Sport Rehabilitation Shanghai University of Sport Shanghai China; ^3^ Head of General Hygiene Department Samara State Medical University Samara Russia

**Keywords:** bone disease, Krüppel‐like factors, osteoarthritis, osteoporosis, osteosarcoma, rheumatoid arthritis

## Abstract

Krüppel‐like factors (KLFs) are crucial in the development of bone disease. They are a family of zinc finger transcription factors that are unusual in containing three highly conserved zinc finger structural domains interacting with DNA. It has been discovered that it engages in various cell functions, including proliferation, apoptosis, autophagy, stemness, invasion and migration, and is crucial for the development of human tissues. In recent years, the role of KLFs in bone physiology and pathology has received adequate attention. In addition to regulating the normal growth and development of the musculoskeletal system, KLFs participate in the pathological process of the bones and joints and are intimately linked to several skeletal illnesses, such as osteoarthritis (OA), rheumatoid arthritis (RA), osteoporosis (OP) and osteosarcoma (OS). Consequently, targeting KLFs has emerged as a promising therapeutic approach for an array of bone disorders. In this review, we summarize the current literature on the importance of KLFs in the emergence and regulation of bone illnesses, with a particular emphasis on the pertinent mechanisms by which KLFs regulate skeletal diseases. We also discuss the need for KLFs‐based medication‐targeted treatment. These endeavours offer new perspectives on the use of KLFs in bone disorders and provide prognostic biomarkers, therapeutic targets and possible drug candidates for bone diseases.

## 1 INTRODUCTION

The Krüppel‐like factor (KLF) family of transcription factors, which means ‘cripple’ in German, is a family of regulators essential for DNA‐binding transcription. Numerous biological functions, such as cell differentiation, proliferation, apoptosis, metabolism and development, depend on it.^1^ KLF was originally discovered to be a crucial developmental gene for Drosophila melanogaster embryo segmentation and mutation.^2^ The first mammalian KLF was first identified in 1993 in erythrocytes and is also known as the erythroid Krüppel‐like factor (EKLF/KLF1).^3^ Up to now, the human genome contains 17 KLF genes (KLF1–KLF17), each of which has a unique tissue distribution pattern and function. These genes are named based on the order.^4^ The KLF family of transcription factors has a wide range of applications and research value in many fields of biology, including gene therapy, tumour therapy and drug development.

Skeletal disorders are a type of inflammatory and degenerative diseases caused by injury or pain in the locomotor organs, mainly affecting the bones, cartilage and joints, including osteoarthritis (OA), rheumatoid arthritis (RA), osteoporosis (OP) and osteosarcoma (OS).^5^, ^6^, ^7^, ^8^, ^9^ Recently, bone diseases have posed a great threat to the health of all human beings, and their incidence and trends are gradually increasing, placing a heavy burden on global health, but appropriate interventions for their management and prevention have still not been proposed.^10^, ^11^, ^12^, ^13^, ^14^, ^15^ In the physiological and pathological progression of bone, the interaction of KLFs‐related proteins with chondrocytes, osteoblasts, osteoclasts and fibroblast‐like synoviocytes (FLS) is crucial.^16^, ^17^, ^18^, ^19^, ^20^ Numerous significant signal pathways, including NF‐kB, Notch, Wnt, TGF‐β and Runx2, can be impacted by the imbalance of KLFs.^21^, ^22^, ^23^, ^24^ Moreover, KLFs may also be employed as noncoding RNAs (ncRNAs) target genes to regulate OS cell proliferation, invasion and migration.^25^, ^26^ Here, we highlight the role of KLFs in bone diseases and discuss the shared molecular mechanism between them by analysing previously published findings. We also talked about the functional importance of drug‐targeted KLFs in the management of bone illnesses, providing a potential area for further research.

## 2 STRUCTURE AND FUNCTION OF KLFS

**FIGURE 1:**
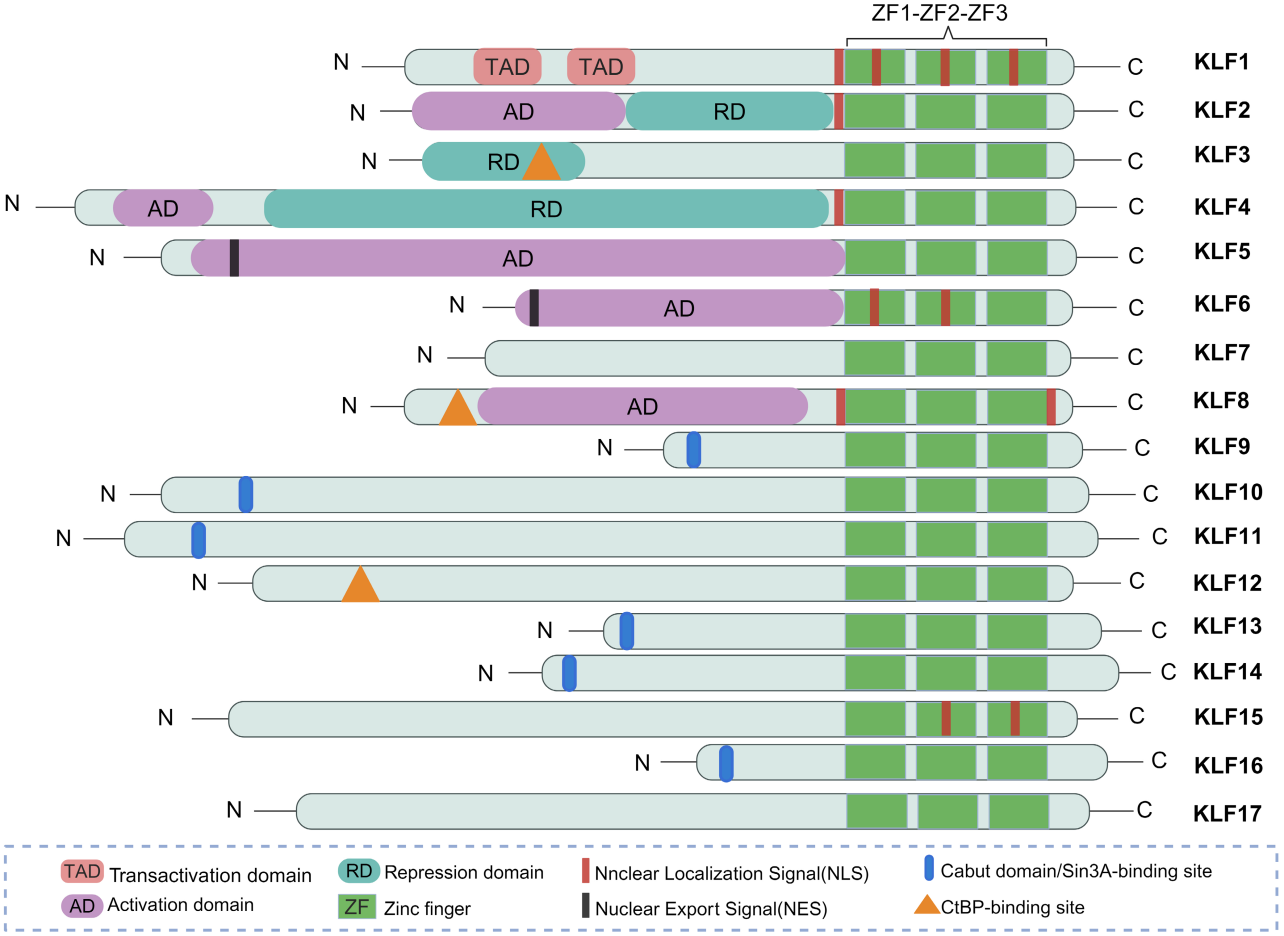
Protein structure of human KLF family members.

**FIGURE 2:**
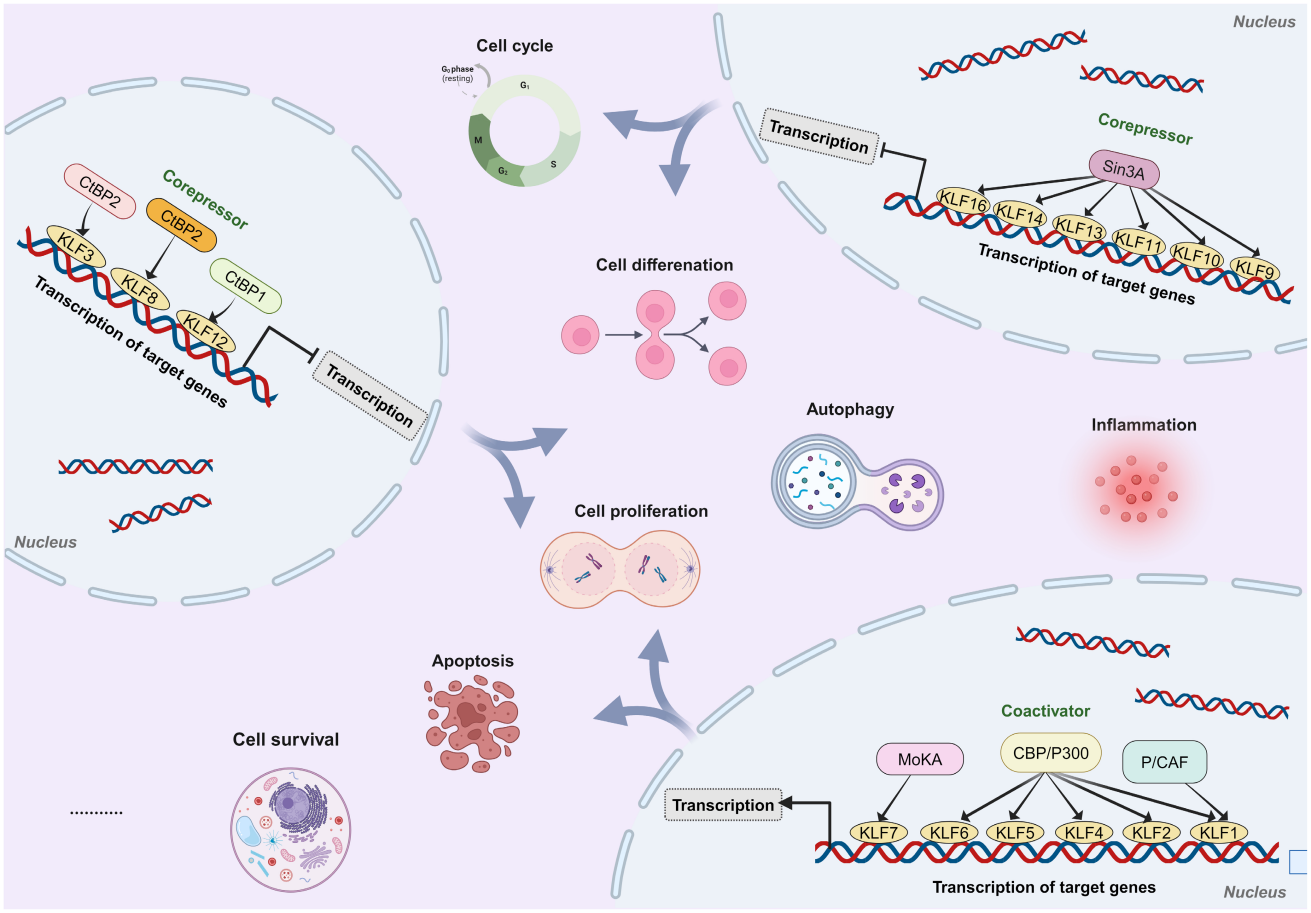
The KLF family is classified based on phylogenetic and structural features: (1) Group 1 contains KLFs 3, 8 and 12, which act as transcriptional repressors by interacting with CtBP; (2) Group 2 contains KLFs 1, 2, 4, 5, 6 and 7, which function as transcriptional activators by binding acetyltransferases; (3) Group 3 members are KLFs 9, 10, 11, 13, 14 and 16, which repress transcriptional activity by interacting with the transcriptional deterrent Sin3A. KLFs are engaged in a variety of physiological and pathological processes, such as cell differentiation, proliferation, growth and apoptosis under normal development or various disease situations, through controlling gene transcription.

KLFs are a group of evolutionarily conserved zinc finger transcription regulators that bind a variety of specific proteins to mediate transcriptional regulation and are involved in regulating physiological processes such as growth, development, differentiation, death, proliferation and embryogenesis.^27^, ^28^ They follow the discovery of Sp1, a mammalian transcription factor, and they are classified as part of the Sp1/KLF family because they contain the same DNA‐binding structural domain (DBD) as Sp1.^29^, ^30^ Up to now, 17 members of the mammalian KLF family (KLF1‐KLF17) have been identified.^28^, ^31^, ^32^ Notably, in 2013 Pei et al. used BLAST sequence similarity searches and gene covariance analysis to identify a newly defined KLF pseudogene in placental mammals and named it KLF18. However, there is still a lack of gene expression evidence for the KLF18 gene, which may be the product of a localized gene duplication of KLF17.^4^, ^33^ All members of the KLF family have three highly conserved Cys2His2‐type zinc finger motifs at their carboxyl termini and each zinc finger structure has a fixed length, with zinc finger structure 1 and zinc finger structure 2 both having 23 amino acid residues and zinc finger structure 3 having 21 amino acid residues.^30^, ^34^, ^35^, ^36^, ^37^, ^38^ In the C‐terminal region of KLF, the three conserved zinc finger motifs express transcriptional repression or activation by binding to DNA sequences enriched in GC boxes or with CACCC homology in target genes.^36^, ^39^, ^40^ Unlike the carboxy‐terminal region, the N‐terminal region of KLF is highly evanescent and often mediates protein–protein and protein‐DNA interactions. In addition, the non‐DNA‐binding region/the N‐terminal region is unique, with transactivation, activation and repressor domains, which in turn exhibit functional variability and diversity by binding to different co‐activators, co‐repressors, modifiers and other specific transcription factors^29^, ^30^, ^41^ (Figure 1). The KLF family is typically divided into three groups based on phylogenetic and structural characteristics. Members of group 1 include KLF3, KLF8 and KLF12, which have ‘PXDLS’ sequence binding sites that interact with C‐terminal binding proteins (CtBPs) to act as transcriptional blockers.^31^, ^40^, ^42^, ^43^, ^44^, ^45^ The transcriptional activators KLF1, KLF2, KLF4, KLF5, KLF6 and KLF7 belong to group 2 and bind acetyltransferases.^31^, ^46^, ^47^, ^48^ KLF 9, KLF10, KLF11, KLF13, KLF14 and KLF16 are members of group 3, and they all possess the α‐helical structure ‘AA/VXXL’ that interacts with the transcriptional blocker Sin3A to suppress transcriptional activity.^31^, ^49^, ^50^, ^51^, ^52^, ^53^, ^54^ Moreover, KLF15 and KLF17 have not been classified because they are phylogenetically more distantly related and the protein interaction motifs have not been identified yet^30^, ^31^ (Figure 2).

Biological and pathological processes regulated by KLF transcription factors continue to be discovered. A wide range of human illnesses, including cancer, cardiovascular disorders, immune system disorders, metabolic disorders, digestive disorders and inflammatory disorders, are linked to KLFs.^30^, ^55^, ^56^ The majority of KLFs take a role in tumour biology by converting somatic cells into induced pluripotent stem (iPS) cells and keeping embryonic stem (ES) cells in their pluripotent state.^57^, ^58^, ^59^, ^60^ A growing number of studies have also extended the KLF transcription factor family to musculoskeletal physiology and its related illnesses. From the muscle level, the expressions of KLF2 and KLF4 are up‐regulated in an ERK5‐dependent manner during the process of muscle cell fusion and skeletal muscle differentiation.^61^ KLF10 is activated in response to TGF‐β signalling involved in skeletal muscle fibrosis in chronic muscle injury.^62^ From the perspective of the bone, KLF5 knockdown can delay fracture repair by regulating the Wnt/β‐catenin signalling pathway.^32^ KLF4 regulates normal bone development by regulating the differentiation and migration of chondrocytes, osteoblasts, osteoclasts and vascular endothelial cells.^63^ From the aforementioned, it can be seen that KLFs play an important regulatory role in musculoskeletal growth and development, and impaired musculoskeletal development and maintenance can lead to a wide variety of skeletal disorders, such as OA, RA, OP and OS. In conclusion, these skeletal conditions are linked to the aberrant expression or regulation of KLFs, and further studies could provide more insights into the pathogenesis of these diseases and offer new directions for the development of therapeutic strategies.

## 3 ROLE OF KLFS IN BONE DISEASES

Skeletal disorders affect the development and progression of the skeleton, which in turn involves the joints of the hands, elbows and knees, ultimately leading to motor dysfunction and, in severe cases, disability in the human body. However, their exact cellular and molecular mechanisms remain unclear. Among them, the pathological processes of OA and RA can be regulated by a family of KLF transcription factors, which are largely associated with their inflammatory effects. In addition, KLFs can regulate OP and OS through multiple physiological pathways (Table 1). Thus, targeting KLF transcription factors has the potential to alleviate the development of these diseases.

**TABLE 1:** Expression of KLFs in bone diseases and its related mechanism.

Diseases	KLFs	Expression (↑/↓)	Cell type	Mechanism of action	Pathways	References
OA	KLF2	**↓**	Chondrocytes	Degradation of extracellular matrix, chondrocyte apoptosis, inflammation, oxidative stress	PKA‐RAP1‐MEK‐CREB, Nrf2/ARE, NF‐κB, Notch	22, 79, 80, 84, 93, 94
	KLF4	**↑/↓**	Chondrocytes, meniscus cells, synovial cells	Degradation of extracellular matrix, chondrocyte apoptosis, inflammation	PKA‐RAP1‐MEK‐CREB, TGF‐β1	83, 84, 85, 95, 241
	KLF5	**↑/↓**	ATDC5 chondrocytes	Degradation of extracellular matrix, chondrocyte apoptosis, inflammation	/	86, 88, 89, 90, 91, 96
	KLF10	**↑**	Chondrocytes	Autophagy, chondrocyte proliferation, migration	/	107, 108
	KLF11	**↓**	Chondrocytes	Chondrocyte apoptosis, oxidative stress, endoplasmic reticulum stress	p38 MAPK	^99^
	KLF15	**↓**	ATDC5 chondrocytes	Degradation of extracellular matrix	/	81, 82
RA	KLF2	**↓**	Monocytes	Inflammation	/	121, 122
	KLF4	**↑**	FLS	Survival, proliferation, inflammation, M1 macrophage polarization	NF‐κB, JAK1‐STAT1	127, 128, 130
	KLF7	**↑**	FLS	Proliferation, migration, apoptosis, inflammation	NF‐κB, JNK	^131^
	KLF9	**↓**	MH7A cells	Inflammation	/	^124^
	KLF10	**↓**	FLS	Viability, proliferation, migration, inflammation	NF‐κB	^126^
OP	KLF2	**↓**	Osteoblast, osteoclast	Differentiation	/	156, 157, 162, 163
	KLF3	**↑**	Osteoblast, hFOB1.19 cells	Differentiation	/	^170^
	KLF4	**↑/↓**	mBMSCs	Differentiation, proliferation	Wnt/β‐catenin	16, 63, 180, 183, 191, 242
	KLF7	**↑**	Osteoclast	Differentiation	/	^194^
	KLF10	**↓**	Osteoclast	Differentiation	NF‐κB	17, 24, 166, 243
	KLF15	**↑**	MC3T3‐E1 cells	Differentiation	Wnt	175, 176
OS	KLF2	**↓**	U2OS cells	Proliferation	/	^207^
	KLF3	**↓**	OS cells	Proliferation, invasion, apoptosis	/	^26^
	KLF4	**↑/↓**	MNNG/ HOS cell, OSCs	Proliferation, migration, invasion, apoptosis, OS cells cancer stemness	p38 MAPK, Wnt, Notch, TGF‐β, Hedgehog	219, 221, 234
	KLF5	**↑**	HOS/MG63 cells	Proliferation, migration, invasion	/	^213^
	KLF6	**↓**	MG63 cells	Proliferation, migration, invasion	/	206, 209, 244
	KLF7	**↑**	OS cells	Proliferation, migration, invasion	Wnt/β‐catenin	^214^
	KLF8	**↑**	Saos‐2 cells, OSCs	Survival, OS cells cancer stemness	/	25, 215
	KLF9	**↓**	OS cells	Proliferation, migration, invasion	/	211, 212
	KLF11	**↓**	OSCs	OS cells cancer stemness	/	^240^

*Note*: ↑: up‐regulation; ↓: down‐regulation; ↑/↓: The expression is controversial.

### 3.1 KLFs and OA

The role of KLFs in the pathophysiology of OA has increasingly come to light in recent years. The most prevalent pathological characteristics of osteoarthritis, a chronic degenerative and debilitating joint disease, are progressive cartilage destruction, synovitis, subchondral bone sclerosis and osteophyte production.^64^, ^65^, ^66^, ^67^ As the disease progresses, it eventually leads to pain, joint dysfunction and even disability, which seriously affects people's quality of life. The pathogenesis of OA is multifactorial, involving the increase of catabolism and the decrease of anabolism in the extracellular matrix (ECM).^68^ Currently, it has been revealed that several cytokines have a role in the pathogenesis of OA, with interleukin‐1β (IL‐1β), tumour necrosis factor‐α (TNF‐α) and interleukin‐6 (IL‐6) being the most significant inflammatory mediators.^69^, ^70^ These inflammatory cytokines and other traumas stimulate the injured chondrocytes to undergo a series of changes, which leads to a progressive imbalance in intra‐articular homeostasis and the eventual onset of OA.^71^ In 2020, approximately 6,541,000 adults aged 40 years and older worldwide will suffer from OA.^72^ Its prevalence increases with age and OA has become a global health burden.^73^, ^74^ However, to date, there is still no effective strategy to treat OA. Therefore, it is crucial to find novel treatment targets for OA. New evidence suggests that KLFs are differentially expressed in articular cartilage and are mainly involved in ECM degradation, inflammatory response, chondrocyte apoptosis, autophagy and oxidative stress. In conclusion, studies on the function of KLFs in relevant tissues may provide new therapeutic avenues for OA.

#### 3.1.1 KLFs and the degradation of ECM

The current study confirms that some KLF family members play a role in joint tissue development and maintenance of cartilage homeostasis by regulating ECM degradation. An imbalance between anabolic and catabolic metabolism of ECM is a key factor in the development and progression of OA.^75^ The degradation of cartilage ECM is regulated by mainly cartilage matrix‐degrading enzymes (MMPs and ADAMTS), which reduce chondrocyte stability by degrading cartilage ECM, gradually leading to the disappearance of proteoglycans and the degradation of collagen type II (Col II), and ultimately to the complete loss of articular cartilage.^76^, ^77^, ^78^ In vitro and in vivo studies have demonstrated the potential role of KLFs in ECM, involving KLF family members such as KLF2, KLF4, KLF5 and KLF15.

KLF2 and KLF15 are OA chondroprotective factors. KLF2 was significantly down‐regulated in human OA cartilage tissue and IL‐1β‐induced SW1353 human chondrocytes. Overexpression of KLF2 would increase the expression of COL2A1, significantly reduce the expression of matrix metalloproteinases MMPs (MMP3, MMP9 and MMP13) and inhibit the OA rat model of MIA‐mediated ECM degradation.^79^ Meanwhile, another study suggested that overexpression of KLF2 ameliorates the breakdown of ECM by inhibiting the activation of the MMP13 promoter and reducing the degradation of Col II, whereas silencing of KLF2 appeared to have the opposite conclusion.^80^ The use of ML385, an inhibitor of nuclear factor erythroid 2‐related factor 2 (Nrf2), significantly attenuated the inhibitory effect of KLF2 on MMP13, suggesting that the inhibition of MMP13 expression by KLF2 is dependent on the activation of the Nrf2/ARE signalling pathway, but no study has yet identified a protein–protein interaction between KLF2 and Nrf2.^79^ This also implies that there may be other potential mechanisms for KLF2‐mediated promotion of Nrf2 nuclear translocation. Similarly, KLF15 was expressed in primary chondrocytes as well as ATDC5 and SW1353 chondrogenic cell lines, whereas it was significantly under‐expressed in chondrocytes from OA patients. Mechanistically, KLF15 reduced TNF‐α‐induced MMP3 expression by binding to the promoter region of MMP3, which in turn improves ECM degradation.^81^ In addition, KLF15 knockout destabilization of the medial meniscus (DMM) mice showed significant cartilage degeneration and had significantly higher cartilage OA‐histopathological scores than wild type(WT)mice. It also significantly attenuated peroxisome proliferator‐activated receptor γ (PPARγ) expression and up‐regulated pIKKα/β, a disintegrin‐like and metalloproteinase with thrombospondin motifs (ADAMTS) 5 and MMP13 expression, which in turn accelerated OA catabolism.^82^

Currently, there is controversy regarding the role of KLF4 and KLF5 in regulating the ECM in OA. It has been reported that overexpression of KLF4 can attenuate cartilage tissue destruction induced by DMM surgical in rats by up‐regulating the cartilage‐specific genes SOX9 and COL2A1 and decreasing the expression of RUNX2 and MMP13.^83^ In 2022, Kawata and her colleagues discovered that overexpression KLF4 in IL‐1β stimulated various cells (OA chondrocytes, meniscus cells and synovial cells) resulted in reduced expression levels of IL‐6, MMP3, MMP13 and ADAMTS5, and intra‐articular injection of AAV‐KLF4 led to a reduction in the degree of joint injury and mechanical abnormal pain, and significant improvement in the OA Research Society International (OARSI) score, meniscus histopathology score and synovitis score in mice operated with DMM.^84^ Notably, KLF4 as a transcription factor also directly up‐regulated cartilage ECM gene expression (COL2A1, COL11A2, ACAN, COMP and PRG4), whereas treatment with GGTI‐298 (a RAP1 inhibitor) eliminated the above results.^84^ This suggests that the effect of KLF4 on cartilage ECM genes is dependent on the PKA‐RAP1‐MEK‐CREB signalling axis. Besides, KLF4 was suppressed in MiR‐7 promoted OA, whereas KLF4 overexpression reversed the ECM degradation of articular cartilage caused by miR‐7 by activating the TGF‐β1 signalling pathway and increasing the levels of p‐Smad2/3.^83^ According to the results above, KLF4 is an OA repressor. In contrast to the results above, KLF4 was detected in affected regions of model rats with OA, which led to an increase in MMP13 mRNA level. This study has further found that newly produced MMP13 is not primarily caused by a KLF4‐mediated increase in its transcription rate but by the attenuation of escaped mRNA.^85^ KLF4 appears to have a dual impact on the ECM, which may be explained by the fact that it has both activating and repressive structural domains. Here, KLF4 as a transcription factor enhanced the expression of MMP13 protein levels by enhancing the stability of MMP13 mRNA, predicting a complex mechanism of KLF4 to stabilize the ECM. However, its specific role needs to be further investigated. Likewise, KLF5 is up‐regulated in IL‐1β‐induced ATDC5 chondrocytes.^86^ N‐myc downstream‐regulated gene 2 (NDRG2) has been identified as a key biomarker and regulator of OA.^87^ Mei et al.^86^ reported that KLF5 overexpression increased the expression of ECM degradation‐related MMP3, MMP13 and ADAMTS4 levels, decreased collagen II expression and reversed the ameliorative effect of NDRG2 on ECM degradation. KLF5 has also been proved to be involved in cartilage degeneration mediated by EGR1, promoting the expression of MMP9 and MMP13 in chondrocytes and inhibiting the expression of COl2A1.^88^ Consistently, KLF5 inhibited the expression of Col II and aggregates in rabbit articular chondrocytes, leading to a significant reduction in sulfated proteoglycan synthesis.^89^ In addition, MMP9 is positively correlated with the expression of KLF5, which contributes to cartilage degradation.^90^ However, Wang et al. found that KLF5 was markedly down‐regulated in human OA cartilage and IL‐1β‐treated chondrocytes, and overexpression of KLF5 partially reversed the down‐regulation of SOX9, COL2A1 and aggrecan expression levels in OA chondrocytes caused by miR‐153‐3p.^91^ KLF5 appeared to protect against ECM degradation. But this study did not search for other potential miR‐153‐3p target genes, so it is unknown if other target genes for these microRNAs (miRNAs) may affect the role of KLF5. Combined with previous studies, it is reasonable to speculate that KLFs are regulated by miRNAs networks to affect OA. Additionally, it has been suggested that KLF5 may include inhibitory structural domains that are still unidentified, which may allow for a change in its action.

In brief, the available findings partially explain how KLF2, KLF4, KLF5 and KLF15 simultaneously regulate the expression of numerous cartilage matrix‐degrading enzymes in chondrocytes, and it is highly likely that modulation of the activity or expression of the KLFs could be a promising therapeutic modality to ameliorate OA.

#### 3.1.2 KLFs and inflammatory response

There is growing evidence that KLFs play an important role in maintaining chondrocyte activity and homeostasis by regulating the expression of inflammatory factors. A large number of inflammatory mediators, such as TNF‐α, IL‐1β, IL‐6, IL‐8, IL‐15 and IL‐17, appear in the synovial fluid of joints with OA, and these inflammatory factors induce reactive oxygen species(ROS) production and cause oxidative stress, which leads to cartilage matrix damage and ultimately the development of OA.^92^ Below we focus on the specific processes by which KLF2, KLF4 and KLF5 affect OA by regulating the production of pro‐inflammatory cytokines.

KLF2 and KLF4 regulate the expression of inflammatory mediators in OA. KLF2 overexpression significantly down‐regulated IL‐6, an inflammatory gene in joint tissue cells.^84^ Long noncoding RNAs (lncRNA) GAS5 in ATDC5 cells was predicted by bioinformatics analysis to target KLF2, and by up‐regulating KLF2, GAS5 reduced inflammatory damage caused by lipopolysaccharides (LPS).^22^ KLF2 has also been shown to be a target gene of miR‐150, and inhibition of miR‐150 resulted in up‐regulation of KLF2, which exhibited a protective effect on IL‐1β‐induced ATDC5 cells.^93^ Among other things, decreased phosphorylation of p65 and IκBα, as well as the expression of Notch1, Notch2 and Notch3, indicate that the overexpression of KLF2 inactivates the NF‐kB and Notch pathways.^22^, ^93^ Further thorough research is necessary to fully understand the mechanisms of action of these two pathways in relation to how miR‐150/KLF2 and GAS5/KLF2 influence OA. In addition to taking part in inflammatory responses, KLF2 also prevents chondrocytes from experiencing oxidative stress. According to Li et al.'s study, KLF2 was activated when IL‐1β‐induced oxidative stress was weakened by montelukast (an antagonist of cysteinyl leukotriene receptor 1, CysLTR1), which reduced the levels of ROS, malondialdehyde (MDA), antioxidant proteins haem oxygenase‐1 (HO‐1) and Nrf2 and increased the level of superoxide dismutase (SOD) in ATDC5 cells, but this effect was significantly reversed after small interfering RNA (si‐RNA)‐KLF2 treatment.^94^ In addition to KLF2, KLF4 reduces IL‐6 to lessen inflammatory responses.^84^ In IL‐1β‐stimulated chondrocytes, Huang et al. discovered a substantial reduction in the expression of KLF4 and lncRNA MEG3.^95^ Overexpression of KLF4 markedly reversed the up‐regulation of the expression levels of IL‐6, IL‐8 and TNF‐α induced by the knockdown of MEG3, and it also enhanced the viability and migration of chondrocytes.^95^ This suggests that KLF4 can be indirectly targeted by MEG3 to protect chondrocytes from IL‐1β‐induced inflammation, and also reveals a link between KLFs and lncRNA in mediating inflammatory responses.

The trend of KLF5 in OA is opposite to the above. In the OA model induced by DMM and LPS, KLF5 and CXCL11 are highly expressed, and CXCL11 overexpression can not only aggravate the pyroptosis of OA chondrocytes, but also stimulate tissue lesions to increase the levels of IL‐1β and IL‐18 in the inflammatory microenvironment.^96^, ^97^ Knocking down KLF5 can significantly down‐regulate the expression of CXCL11, which reveals a new insight that KLF5 regulates OA.^96^ JASPAR database found that there may be interaction between KLF5 and NDRG2 and the transcriptional inhibition of KLF5 on NDRG2. Indeed, overexpression of KLF5 counteracts the effect of NDRG2 on reducing the release of inflammatory cytokines by up‐regulating the expression of a large number of pro‐inflammatory cytokines (TNF‐α, IL‐6, p‐NF‐κB p65 and Cox‐2).^86^

Overall, the aforementioned findings provide compelling evidence that aberrant expression levels of KLF2, KLF4 and KLF5, important OA regulatory factors, can impact the inflammatory pathological state of OA. KLF5 stimulates inflammation, while KLF2 and KLF4 are negative regulatory factors in OA inflammation.

#### 3.1.3 KLFs and chondrocyte apoptosis

KLFs have been reported to play an important role in the maintenance, reconstitution and renewal of articular cartilage morphology and structure by regulating chondrocyte apoptosis. Apoptosis is an evolutionarily conserved programmed cell death process that is essential for regulating cell growth and development, senescence and maintaining tissue homeostasis. Once apoptosis is dysregulated, it can lead to a range of diseases such as developmental defects, degenerative diseases, autoimmune diseases, neurodegeneration and cancer.^98^ Chondrocyte apoptosis, an important pathological process in OA, leads to a rapid disintegration of the cellular matrix, and these two interact with each other in a vicious circle. To date, studies have confirmed the association of KLFs with chondrocyte apoptosis, mainly related to KLF2, KLF5 and KLF11.

KLF2 and KLF11 slow down OA by inhibiting chondrocyte apoptosis. The activity of KLF2 was enhanced in the process that montelukast attenuated chondrocyte apoptosis.^94^ The role of KLF2 in this process was to reduce the proportion of chondrocyte apoptosis, decrease the expression of pro‐apoptotic Bax, APAF‐1, Cleaved caspase 3 and Cleaved PARP, and increase the expression of the anti‐apoptosis protein B‐cell lymphoma 2 (Bcl‐2).^94^ Similarly, Gao et al. found that KLF2 overexpression significantly reduced the percentage of IL‐1β‐induced apoptosis in SW1353 cells. Mechanistically, KLF2 overexpression activates Nrf2/ARE signalling to promote the transcription of Nrf2 target genes, thereby inhibiting chondrocyte apoptosis.^79^ Besides, KLF2 was involved in NF‐κB and Notch signalling pathways to ameliorate LPS‐induced inflammatory reaction and apoptosis in ATDC5 chondrocytes.^22^ The above results show that KLF2 is a key factor in the regulation of OA metabolism and its changes lead to pathological changes in OA. Of course, KLF2 can also be used as a target for molecular inhibitors to ameliorate OA. Same as KLF2, KLF11 was decreased in OA patients and IL‐1β‐stimulated articular chondrocytes.^99^ Overexpression of KLF11 can reverse the decrease in Bc1‐2 and the increase in Bax caused by IL‐1β treatment. Furthermore, KLF11 overexpression also increased the expression of SOD1, SOD2, peroxiredoxin 1 (Prdx1) and peroxiredoxin 4 (Prdx4) and significantly decreased the expression of endoplasmic reticulum stress (ERS)‐related signalling pathways (CHOP, ATF‐6 and GRP‐78). Interestingly, KLF11 inhibited the expression of p38 and p‐p38 in the MAPK signalling pathway.^99^ This suggests that KLF11 slows down chondrocyte apoptosis, oxidative stress and ERS in OA by inhibiting the p38 MAPK signalling pathway.

In contrast, KLF5 promotes chondrocyte apoptosis in OA. Some researchers discovered that KLF5 overexpression reversed the inhibition of chondrocyte apoptosis by overexpression of NDRG2, a differentially expressed gene common to human OA and rat OA models. Overexpressing NDRG2 or overexpressing both NDRG2 and KLF5 in IL‐1β‐stimulated ATDC5 chondrocytes, it was found that the expression of Bcl‐2 was reduced in the group overexpressing both NDRG2 and KLF5 compared with the group overexpressing NDRG2 alone and that the expression of the Bax, Cleaved caspase 3/Caspase 3 and Cleaved PARP/PARP expression increased, as well as TUNEL staining showed a dramatic increase in the number of positive cells.^86^ It is evident that excessive KLF5 expression induces apoptosis and results in decreased chondrocyte viability.

In conclusion, the expression of KLF2, KLF5 and KLF11 is closely related to chondrocyte apoptosis, which is very likely to develop into potential biomarkers of OA and can also be served as targets for OA medication therapy, which still needs to be further clarified.

#### 3.1.4 KLFs and autophagy

In recent years, more and more studies have found that KLF family members participate in the progress of many diseases through autophagy.^100^, ^101^, ^102^ Autophagy is a conservative lysosomal degradation process, which is very important for cell homeostasis and adaptation to stress.^103^, ^104^ People gradually realize that autophagy plays an important role in the degeneration of OA. Abnormal autophagy and mitochondrial autophagy will destroy the balance of bone metabolism and play a huge role in maintaining bone metabolism.^105^, ^106^ At present, KLF10 has been proved to influence OA through controlling autophagy.

A recent study found that high expression of KLF10 was detected in human OA cartilage and mouse OA cartilage.^107^ Notably, KLF10 was also up‐regulated in senescent chondrocytes. Down‐regulation of KLF10 not only restored the impaired mitochondrial autophagic flux, but also attenuated tert‐butyl hydroperoxide (TBHP)‐induced chondrocyte senescence by promoting mitochondrial autophagy, facilitated cell proliferation and inhibited senescence‐associated secretory phenotypes (SASPs), including the secretion of IL‐6, CXCL10, MCP1 and MMP3. Interestingly, the application of an autophagy inhibitor to block autophagy revealed that the inhibitory effect of the knockdown of KLF10 on chondrocyte senescence was eliminated. Surprisingly, co‐knockdown of KLF10 and Bcl2‐interacting protein 3 (BNIP3) resulted in a decrease in COL2A1 and LC3II/I and an increase in the expression of MMP13, p62, p16 and p21, as seen in the suppression of autophagic flux. This seems to indicate that KLF10 regulates autophagy by regulating the expression of BNIP3.^107^ Moreover, another study reported that KLF10 overexpression accompanied by up‐regulation of Acvr1 gene expression and down‐regulation of Inhbb gene expression resulted in a significant reduction in chondrocyte proliferation and migration, promoting OA.^108^ Although less progress has been reported on the involvement of KLFs in the regulation of OA through autophagy, this offers a direction for future research.

### 3.2 KLFs and RA

KLFs have been reported to be closely associated with the development of RA, which is categorized as a chronic systemic immune joint disease with an erosion of the joints, and the main pathological manifestations of which are massive immune cell infiltration, synovial inflammation and hyperplasia as well as the destruction of the articular cartilage and bone.^109^, ^110^, ^111^, ^112^ RA has become a prevalent disease throughout the world, and with the course of the disease, it can ultimately involve a wide range of tissues, organs and systems.^113^, ^114^, ^115^, ^116^ FLS and macrophages play an important role in the pathogenesis of RA.^117^ Synovial macrophages secrete inflammatory cytokines such as TNF‐α and IL‐1β, which activate FLS. FLS is then engaged in the local expansion of macrophages by up‐regulated IL‐6, creating a vicious cycle. Activated FLS also produce MMPs, which in turn lead to cartilage degeneration and osteoclastogenesis.^117^, ^118^, ^119^ Consequently, it appears that focusing on FLS, a unique cell type, will enhance the clinical outcome of RA. Emerging evidence suggests that several KLFs, mainly including KLF2, KLF4, KLF7, KLF9 and KLF10, are involved in RA by regulating inflammatory responses and FLS proliferation and migration.

#### 3.2.1 KLFs and inflammation

The most significant contributing component to the pathophysiology of RA is inflammation. The activation of endothelial cells and macrophages during inflammation is facilitated by inflammatory mediators, which are up‐regulated during RA inflammation and ultimately contribute to the activation and dysfunction of RA‐fibroblastoid synovial cells (RA‐FLS).^120^ The body of current research indicates that KLFs are important for the pathophysiology of RA. They can affect the inflammatory response in RA and play a function in controlling of synovial membrane structure and tissue destruction. The following is a review of how KLFs leads to the progression of chronic inflammation in RA.

KLF2, KLF9 and KLF10 were proven to inhibit the expression of inflammatory mediators in RA. According to Das, KLF2 is low expressed in RA mice and RA patients. Compared with KLF2 WT mice, KLF2 knockout mice developed more severe paw swelling and synovitis after arthritis induced by K/BxN serum, and cartilage and bone erosion were significant, which aggravated the pathological changes of RA. The levels of inflammatory cytokines (MCP1, IL‐1β, IL‐6 and TNF‐α) were significantly increased. Interestingly, KLF2 was negatively correlated with MMPs, especially MMP9 expression.^121^ Specifically, deletion of KLF2 significantly increased the accumulation of histone acetylation markers (H3K9Ac and H4K8Ac) and histone acetyltransferase (HAT, P300 and PCAF) at the transcription start site (TSS) of MMP9, thus promoting the expression of MMP9.^121^ Another study by Das also confirmed that overexpression of KLF2 down‐regulated the expression of MMP9, thus reducing joint inflammation and cartilage injury.^122^ Following the foregoing, KLF9 is down‐regulated in MH7A cells (human rheumatoid arthritis fibroblasts) induced by TNF‐α, while the enhancement of KLF9 can inhibit the release of pro‐inflammatory factors (IL‐1β, IL‐6, IL‐8, iNOS and COX‐2).^123^, ^124^ Tripartite motif containing gene 33 (TRIM33) is a member of TRIM family containing E3 ubiquitin ligase, which can inhibit the secretion of inflammatory factors by synovial fibroblasts.^124^, ^125^ The luciferase report found that the luciferase activity in MH7A cells transduced by TRIM33‐WT and si‐KLF9 decreased, whereas the enrichment of TRIM33 was observed after adding anti‐KLF9.^125^ The above results suggest that overexpression of KLF9 increases the enrichment of TRIM33, while down‐regulation of KLF9 weakens the inhibitory effect of TRIM33 on inflammatory response, indicating that KLF9 may partially inhibit inflammation in RA by positively regulating TRIM33.^124^ Furthermore, Wang et al.^126^ discovered that KLF10 expression in RA‐FLS was dramatically reduced and that KLF10 may activate PDZ and LIM domain‐containing protein 2 (PDLIM2), which have anti‐inflammatory and anti‐proliferative properties. Knocking out KLF10 not only undid the effect of PDLIM2 on restraining the release of inflammatory factors (TNF‐α, IL‐1β, IL‐6, COX2 and iNOS) from RA‐FLS but also boosted the expression of NF‐κB pathway‐related proteins (p‐IκBα and p‐NF‐κB).^126^ KLF10/PDLIM2 may repress inflammation in RA‐FLS by controlling the NF‐κB pathway.

KLF4 and KLF7, on the other hand, support the inflammatory reaction in RA. It is reported that TNF‐α induced RA synovial cells exhibited significant levels of KLF4 expression, and luciferase assay revealed that KLF4 trans‐activated the inflammatory factor iNOS promoter and encouraged its expression.^18^ Luo's research also confirms that KLF4 is highly expressed in synovial tissue and FLS of RA patients, and directly activates the IL‐6 promoter and interacts with NF‐κB in response to TNF‐α‐mediated activation of FLS in RA.^127^ Consistent with this, there is evidence that the expression of IL‐6, IL‐1β, TNF‐α and MMP‐13 in the synovial cells of mice with collagen antibody‐induced arthritis (CAIA) knocked out by KLF4 is much lower than that of the control mice, but the severity of collagen‐induced arthritis (CIA) in mice expressing KLF4 is higher than that in mice injected with blank control vector.^128^ In the synovium of mice and humans, the deletion of KLF4 also decreased the breakdown of type I collagen and down‐regulated the production of MMPs (MMP2, MMP9, MMP12 and MMP13) and Bcl‐2 mRNA.^128^ It is thought‐provoking, this study used ribonucleic acid (RNA)‐guided endonuclease (RGEN) to produce C57BL/6 background KLF4 knockout mice. The skin phenotype of KLF4 RGEN knockout mice is different from that of classical knockout mice using homologous recombination system.^129^ Whether different knockout methods will affect the expression of KLF4 on regulating FLS and inflammatory mediators is unknown, and the exact mechanism of this difference should be clarified through further research. In addition to regulating inflammatory response, KLF4 also takes a role in RA by regulating macrophage polarization. An in vitro study found that overexpressing KLF4 dramatically boosted the levels of the inflammatory cytokines TNF‐α, IL‐6 and IL‐1β as well as CD86, a macrophage M1 marker. Due to the overexpression of KLF4, JAK1, p‐JAK1, STAT1 and p‐STAT1 expression levels also rose. More precisely, KLF4 regulates the activation of the JAK1‐STAT1 signal and its degree of phosphorylation, which increases the polarization of M1 macrophages and exacerbates RA.^130^ Consistent with KLF4, the expression of KLF7 in synovial tissue of rats with adjuvant‐induced arthritis (AIA) increased significantly.^131^ KLF7 knockout inhibited the release of inflammatory factors (IL‐1β, IL‐6 and IL‐17A) in RA‐FLS by blocking the phosphorylation of NF‐κB p65 and JNK.^131^ Conversely, overexpression of KLF7 can enhance the activation of NF‐κB and JNK pathways, leading to inflammatory reactions in RA‐FLS.

The previous results demonstrated that KLF2, KLF4, KLF7, KLF9 and KLF10 can influence the pathological alterations of RA by modulating the inflammatory response. However, the specific mechanism needs to be further explored, and it is necessary to deeply explore the signal pathways associated with KLFs and RA and the interaction of ncRNAs.

#### 3.2.2 KLFs and FLS proliferation, migration and invasion

FLS has unique aggressive behaviours, which play a positive role in the progress of RA. FLS not only responds to the inflammatory environment,^132^ but also has an activated and invasive phenotype, which plays a role independently of inflammatory stimulation.^133^ At present, some researchers have found that KLF7, KLF9 and KLF10 can regulate the proliferation, migration and invasive phenotype of RA‐FLS, and it is very important to explore their specific mechanisms to reverse the deterioration of RA.

KLF7 not only enhanced the inflammatory reaction in RA but also boosted the proliferation of FLS. Compared with the control group, the expression of KLF7 mRNA and protein in synovial tissue of AIA rats was significantly increased.^131^ Activation of NF‐κB and JNK signal pathways is very important to accelerate the progress of RA.^133^, ^134^ KLF7 can promote the proliferation of FLS by activating NF‐κB and JNK signalling pathways, and up‐regulate the expression of MMPs (MMP1, MMP3 and MMP13), an important regulator of FLS invasive phenotype. They also discovered that KLF7 was a potential target of miR‐9a‐5p and their expression was negatively associated.^131^ This manifests that inhibiting KLF7 by inducing miR‐9a‐5p seems to improve RA.

In RA, KLF9 has a biologically different function than KLF7. For instance, suppression of miR‐130a‐3p and miR‐218‐5p causes KLF9 expression to increase, which in turn reduces RA‐FLS proliferation, migration, invasion and cell survival.^123^, ^135^ Interestingly, silencing miR‐218‐5p to ameliorate oxidative stress and promote apoptosis and autophagy in RA was reversed under KLF9‐deficient conditions.^135^ It follows that KLF9 may inhibit RA‐FLS proliferation by encouraging apoptosis and autophagy. Additionally, KLF9 positively regulated TRIM33 expression to prevent TNF‐α induced abnormal proliferation and migration of MH7A cells.^124^ Same as KLF9, inhibition of KLF10 can eliminate the suppressive effect of PDLIM2 on RA‐FLS, which shows that the activity of FLS is significantly increased, FLS migration is increased, the expression of Bcl‐2 is increased, the expression of apoptosis‐related proteins (Bax, Cyto‐C and Cleaved caspase 3) is decreased, and the expressions of inflammatory factors and MMPs (MMP2 and MMP9) are markedly increased. Further studies have proved that KLF10 can negatively impact the expression of NF‐κB pathway‐related proteins p‐IκBα and p‐NF‐κB, thus affecting the cell viability, proliferation and migration of FLS.^126^

The above conclusion indicated that KLF7, KLF9 and KLF10 are closely related to the proliferation, migration, invasion and cell viability of RA‐FLS, which can help us better predict the pathological status of diseases to a certain extent. It involves microRNA targeted regulation of KLF expression, which seems to be a new target for treating RA, but the specific mechanism remains to be revealed.

### 3.3 KLFs and OP

Nowadays, it has been established that KLFs are involved in controlling the pathophysiology of OP. OP is a prevalent systemic metabolic bone disease, defined primarily by loss of bone mass and deterioration of the bone microstructure, which ultimately results in bone fragility and an increased risk of fracture.^136^, ^137^, ^138^, ^139^ To maintain stable bone mass and mineral homeostasis, the normal bone remodelling process necessitates a coordinated balance between bone formation by osteoblasts and bone resorption by osteoclasts.^140^, ^141^, ^142^ Once osteoclast‐mediated bone absorption surpasses osteoblast‐mediated bone formation, it will lead to an imbalance of bone remodelling and bone loss, which will eventually evolve into OP.^143^, ^144^, ^145^, ^146^ With the ageing of the world population, OP has become a global public health burden.^145^, ^147^, ^148^, ^149^ The latest study points out that several KLF transcription factors have a role in modulating bone formation and resorption as well as bone health and disorders.^19^, ^150^, ^151^, ^152^ Hence, determining the probable link between KLFs and osteoblasts and osteoclasts for the onset and progression of OP is crucial.

#### 3.3.1 KLFs and bone formation

KLFs regulate osteoblast differentiation, which plays a role in the pathological development of OP. Osteoblasts secrete extracellular matrix proteins to reconstruct bone and bone matrix mineralization, and their differentiation is crucial to bone formation and the maintenance of bone remodelling homeostasis, whose disruption results in many bone‐related disorders.^153^, ^154^, ^155^ Existing research has shown that KLFs, primarily KLF2, KLF3, KLF4, KLF10 and KLF15, play momentous roles in adjusting osteoblast differentiation.

KLF2 and KLF10 inhibit OP development as positive regulators of bone metabolism and bone differentiation. New research demonstrated that overexpression of KLF2 enhanced the formation of bone nodules and drastically up‐regulated the expression of the genes that indicated osteoblast differentiation (RUNX2, ALP, OSX, OCN and BSP).^156^, ^157^ RUNX2 is a key central regulator of osteoblast differentiation, matrix synthesis and mineralization.^157^, ^158^, ^159^, ^160^ By interacting with RUNX2 in osteoblasts, KLF2 increases the activity of genes unique to osteoblasts, hence boosting osteogenic differentiation.^157^ Meanwhile, Kim et al. discovered that KLF2 knockdown altered the capacity of the gene activator interferon regulatory factor 2‐binding protein 2 (IRF2BP2) to promote osteoblast development.^156^, ^161^ KLF2 not only positively controls the expression of genes relevant to osteoblast differentiation but also affects osteoblast differentiation through mediating autophagy process. Maity et al. discovered that after overexpressing KLF2 in dental pulp‐derived stem cells (DPSCs), the number of autophagosomes and the expression of autophagy‐related molecules (LC3B‐II: LC3B‐I ratio, BECN1, ATG5 and ATG7) rose considerably. The levels of autophagy regulatory molecules and markers specific for osteoblast differentiation (RUNX2, BGLAP and SPARC) reduced after the knockdown of KLF2.^162^ Another study by Maity found that KLF2 could be up‐regulated by a non‐steroidal phytoestrogen (ferutinin), thus enhancing the binding efficiency of KLF2 with ATG7 promoter region and promoting autophagy and osteoblast differentiation.^163^ Similarly, KLF10 is a gene related to the volumetric cortical bone mineral density, and KLF10 knockout mice show a decrease in the number of osteoblasts, bone trabecular defects, a reduction in sclerotin and a decrease in the mineralization ability of osteoblasts.^24^, ^151^, ^164^, ^165^, ^166^, ^167^ On the one hand, KLF10 may accelerate bone defect in OP by activating Wnt signal transduction and promoting osteoblast differentiation.^168^ On the other hand, KLF10 may favourably influence the transcription activity of Runx2 via the ubiquitin/proteasome pathway or by regulating cytokines TGF‐β1 and bone morphogenetic protein 2(BMP2), thus promoting osteoblast differentiation, bone matrix formation and mineralization.^24^ Additionally, another study observed that KLF10 expression was significantly down‐regulated in OP patients and ovariectomy‐induced OP in rats, while down‐regulation of KLF10 decreased the expression of markers associated with osteogenesis (RUNX2, ALP, OCN and OSX), which ultimately prevented osteogenic differentiation in OP and aggravated the process of OP.^17^, ^169^ In summary, KLF2 and KLF10 are positive regulators of osteoblast differentiation and play critical roles in skeletal development and the preservation of bone metabolic homeostasis.

Unlike KLF2 and KLF10, KLF3 and KLF15 act as negative regulators of bone formation to promote OP. KLF3‐specific knockout can promote the differentiation of human osteoblasts (hFOB1.19 cells) and alkaline phosphatase (ALP) activity.^170^ Another in vivo study also confirmed that mice with knockout of endothelial‐specific KLF3 showed an increase in the number of CD31^hi^EMCN^hi^ endothelium, bone volume, ALP‐positive osteoprogenitors and bone surface OCN osteoblasts.^171^ JunB is a key factor in regulating OP, which is involved in regulating angiogenesis and positively regulating osteoblast differentiation.^172^, ^173^, ^174^ According to Yang et al.,^171^ KLF3 directly suppressed osteogenic differentiation by directly inhibiting the transcription of its downstream gene JunB, and this process can be counteracted by using a human high bone mass (HBM)‐associated lncRNA Reg1cp mutant in combination with KLF3. Ophiopogonin D, a natural compound, has a similar function to the mutant Reg1cp, which can increase the expression of JunB by inhibiting the function of KLF3, and then play an anti‐OP role.^171^ The regulatory impact of KLF15 on OP is similar to that of KLF3. KLF15 was up‐regulated in bone tissue of rats with glucocorticoid (GC)‐induced OP (GIO) and in mouse embryonic osteoblast preosteoblastic (MC3T3‐E1 cells) exposed to dexamethasone (Dex).^175^, ^176^ Exogenous overexpression of KLF15 attenuated the expression of bone formation‐related factors (ALP, OPN, OCN, RUNX2 and Osterix) and effector TCF4 downstream of Wnt signalling and promoted ROS generation, caspase 3 activity and expression of apoptosis‐associated protein p‐p66^Shc^ induced by Dex, which in turn exacerbated osteogenesis inhibition in OP.^176^ Tanshinol, as a polyphenolic compound, can play an anti‐OP role by enhancing bone formation in vivo and in vitro.^177^, ^178^ In the absence of KLF15, the effect of tanshinol on slowing down bone injury will be strengthened, and blocking the activation of KLF15 mediated by GIO can promote osteogenic differentiation.^175^, ^176^, ^179^ Therefore, KLF3 and KLF15 play an important role in the pathogenesis of OP, and they have great potential as drug therapy targets.

Besides the controversy in OA, there are different views on the effect of KLF4 on osteoblast differentiation in OP. The role of KLF4 in OP is mainly related to the regulation of bone formation and bone development. In an in vivo study, KLF4 was deleted by using the Sp7‐Cre deletion strain, and it was found that mice showed reduced bone mineralization and delayed ossification.^16^ Deletion of KLF4 inhibited osteoblast differentiation in mouse bone marrow stromal cells (mBMSCs), which was manifested by the decrease of matrix mineralization and the reduction of osteogenesis‐related markers OPN, SP7, RUNX2 and Bglap.^16^ Yu and colleagues further found that KLF4 itself could not trigger osteoblast differentiation, but rather, it was affected by the BMP2 signalling pathway and regulated osteoblast differentiation in conjunction with osteogenesis‐rich transcription factors (RUNX, DLX, MADS and CEBPE).^16^ However, puzzlingly, KLF4‐deficient mice generated using the Col1α‐Cre strain exhibit increased bone mass and the expression of osteogenic markers (RUNX2, ALPL and BSP) was detected to be strongly enhanced in KLF4 conditional knockout osteoblasts.^180^ The use of different Cre mouse strains shifted the role of KLF4, with the Sp7‐Cre mouse strain characterizing early osteoblast precursors, that is, mainly targeting immature osteoblasts, whereas Col1α‐Cre‐mediated deletion of the KLF4 gene occurs more efficiently in mature osteoblasts.^181^, ^182^ This seems to suggest that KLF4 is beneficial during the early stages of osteoblast development but inhibits osteogenesis during the late stages. Overexpression of KLF4 adversely influenced osteoblast proliferation, reduced colony formation effectiveness and cell survival in bone marrow mesenchymal stem cells (BMSCs), in addition to altering osteoblast differentiation.^183^ In general, KLF4 appears to have varied functions at various phases of bone formation and development. However, more investigation is required to determine the precise role of KLF4 during the whole osteoblast differentiation stage.

#### 3.3.2 KLFs and bone resorption

Research already conducted demonstrates that KLFs influence OP via controlling osteoclast differentiation. Osteoclasts are the only cells that can absorb bones. They dissolve collagen and other matrix proteins during bone absorption by secreting acids and proteolytic enzymes and play a central role in physiological bone remodelling and pathological bone damage.^184^, ^185^, ^186^ Its mediated rate of bone resorption exceeding the rate of bone formation is a key factor contributing to bone loss and increased fracture risk in OP.^187^, ^188^, ^189^ Currently, several KLFs, primarily KLF2, KLF4 and KLF7, can control the progression of OP by regulating osteoclast differentiation.

In osteoclast differentiation, KLF2 and KLF4 have a regulatory function that is detrimental. KLF2 regulates autophagy, which has an impact on osteoclast differentiation. In RAW264.7 cells (mouse macrophage cell line), Laha et al. discovered that overexpression of KLF2 decreased the creation of autophagic vesicles and the expression of BECN1. Specifically, KLF2 inhibited BECN1 by reducing the recruitment of histone acetylation marks (H3K9Ac and H4K8Ac), resulting in the inhibition of autophagy and osteoclast differentiation.^190^ In the same year, another study verified that overexpression of KLF2 significantly reduced the number of osteoclasts and suppressed the expression of osteoclast differentiation marker genes (c‐Fos, NFATc1 and TRAP), and, conversely, down‐regulation of KLF2 enhanced osteoclast differentiation.^156^ Prior reports have reported that the regulation of KLF4 on osteoblast differentiation is still debatable and that KLF4 is a transcriptional repressor during osteoclast formation.^180^ KLF4 inhibits 1,25 (OH)_2_D_3_‐induced osteoblastic differentiation mainly by negatively regulating the expression of osteoclast differentiation factor (RANKL). Specifically, KLF4 strongly inhibits 1,25 (OH)_2_D_3_‐induced RANKL promoter activity and KLF4 competes with the vitamin D receptor (VDR) to block VDR binding to the RANKL promoter region, thereby attenuating 1,25 (OH)_2_D_3_‐VDR signalling‐induced RANKL expression.^180^ Furthermore, KLF4 is an inhibitor of the Wnt‐β‐catenin signalling pathway, and the transgenic mice expressing KLF4 have severe bone deformity and bone loss, which is very similar to the bone phenotype of mice lacking β‐catenin in osteoblasts.^63^, ^191^, ^192^ It appears to suggest that KLF4 controls RANKL expression mediated by osteoblasts, presumably via the Wnt‐β‐catenin signalling pathway.^180^ There appears to be a time‐dependent regulatory effect of KLF4 on osteoblast differentiation, but whether the specific effect of KLF4 on osteoclast differentiation also correlates with osteoclast maturity is not yet known, and thus, further studies are needed to fully understand the precise molecular mechanisms of KLF4 function in osteoclast differentiation.

The regulatory effect of KLF7 on osteoclasts is different from that of the above KLF members. Chen et al. have discovered that KLF7 can affect the bone mineral density (BMD) of patients through sequencing and bioinformatics, which seems to make it possible that KLF7 will be a target for the treatment of OP.^193^ A study in 2022 showed that KLF7 was low expressed in people with high BMD, and KLF7 was an upstream regulator of HO‐1.^194^ Knockdown of KLF7 impaired osteoclast differentiation, whereas high expression of KLF7 markedly reduced the expression of HO‐1, a negative regulator of osteoclast differentiation, and increased the expression levels of osteoclast marker proteins (TRAP, NFAT2 and CTSK).^194^, ^195^ In short, KLF7 promotes osteoclast differentiation by negatively regulating the expression of HO‐1, thus aggravating OP. Theoretical support for clinical treatment and drug development of OP will come from further investigation into the potential molecular mechanism of KLF7 in OP since there are currently few studies on how KLF7 regulates OP.

### 3.4 KLFs and OS

KLFs are involved in controlling the pathogenic phase of OS, according to studies. OS is a malignant primary bone tumour with a poor prognosis.^196^ Clinically speaking, it is mostly shown by pain, swelling and muscle atrophy in the afflicted bones, which limits joint mobility and, occasionally, causes pathological fractures.^197^, ^198^, ^199^, ^200^ DNA methylation, ncRNAs regulation, histone modification and DNA mutation are the major processes of osteosarcoma. There is presently no viable therapeutic method for OS because of its enormous genetic complexity and high degree of heterogeneity.^201^ Surprisingly, KLFs have a function in regulating the proliferation, migration and invasion of OS cells as well as affecting OS cancer stem cell‐like phenotype, and the main KLF family members involved are KLF2, KLF3, KLF4, KLF5, KLF6, KLF7, KLF8, KLF9 and KLF11.

#### 3.4.1 KLFs and OS proliferation, migration and invasion

KLF2, KLF3, KLF6 and KLF9 are all considered potential tumour suppressors.^202^, ^203^, ^204^, ^205^, ^206^ KLF2 is reported to be a cell cycle‐related gene that inhibits OS cell proliferation by inducing GO/G1 phase cell cycle arrest and causing apoptosis.^207^ Knockdown of KLF2 increased cell viability in U2OS cells (human OS cells), and conversely, high expression of KLF2 exerted pro‐apoptotic effects and inhibited proliferation through activation of cell cycle inhibitory genes p15 and p21.^208^ Similar to KLF2, KLF3 impacts the cell cycle and is only weakly expressed in human soft tissue sarcomas and OS tissues.^26^, ^205^ Overexpression of KLF3 up‐regulates p21 in OS cells and counteracts the rise in Cyclin D1 expression mediated by RNA demethylase (FTO), which prevents OS cells from proliferating and invading and instead encourages apoptosis.^26^ Interestingly, KLF6 also inhibits OS by affecting the cell cycle. Zhu et al.^206^ found that overexpression of KLF6 up‐regulated the expression of p21 in MG63 cells (human OS cells) while decreasing the expression of bcl‐2 and MMP‐9. On the contrary, KLF6 knockout promoted the survival, proliferation and invasion of MG63 cells. Research conducted by Liu also supported this. They have discovered further that KLF6 is the downstream target of ncRNAs (circMTO1 and miR‐630), and it can inhibit the proliferation, migration and invasion of OS cells and promote apoptosis.^209^ Many members of the KLF family participate in the complex regulatory network of ncRNAs, which also includes KLF9. For example, KLF9, as the downstream target of circ_0078767, is up‐regulated, thus inhibiting the proliferation, migration, invasion and epithelial‐mesenchymal transition (EMT) of OS cells and promoting cell apoptosis.^210^ Likewise, KLF9, located downstream of miR‐652, is down‐regulated by it to promote OS cell proliferation and invasion.^211^ Importantly, KLF9 can be regulated downstream of ncRNAs and directly affect the expression of miRNAs. For instance, KLF9 as an upstream regulatory molecule of miR‐338‐3p directly enhances the activity and expression of miR‐338‐3p, which exerts an inhibitory effect on the proliferation, migration and invasion of OS cells.^212^ Therefore, the relationship between KLFs and ncRNAs in OS progression deserves further exploration.

KLF5, KLF7 and KLF8 also participate in the regulation of complex ncRNAs networks, but they all contribute to the development of OS. It is reported that KLF5, KLF7 and KLF8 are strongly expressed in OS.^213^, ^214^, ^215^ Overexpression of KLF5 significantly up‐regulated the expression of miR‐487a in HOS and MG‐63 cells (OS cell lines) but decreased the expression of NK3 homeobox 1(NKX3‐1) with tumour inhibitory properties.^213^ This indicated that KLF5 promoted OS by directly regulating microRNA. Notably, ML264, a small molecule inhibitor of KLF5, can induce a cell cycle halt in the G0/G1 phase and damage OS cell migration, invasion and EMT.^216^ NcRNAs also regulate KLF5, KLF7 and KLF8, in addition to KLF5. For instance, lncRNA KCNQ1OT1 located upstream of KLF7 can up‐regulate the expression of KLF7 by inhibiting the expression of miR‐3666, thus promoting the proliferation, migration and invasion of OS and activating Wnt/β‐catenin signals.^214^ Similarly, KLF8 was down‐regulated by miRNA‐1236‐3p, which inhibited the proliferation of OS cells and induced apoptosis.^25^ It is noteworthy that both KLF8 and KLF2 can influence the cell cycle, but their effects are opposite.^207^, ^217^ Lin et al. knocked out KLF8 in Saos‐2 cells (human OS cells) and observed obvious cell cycle arrest in the G0/G1 phase, and cell invasion was also inhibited.^217^ Obviously, KLF family members have a completely distinct impact on OS by regulating the cell cycle.

The many regulatory roles of KLF4 are discussed in OA and OP, and its function in OS is also disputable. At present, KLF4 is mainly considered to play the role of proto‐oncogene in OS, which can propel the progress of OS by inhibiting apoptosis and promoting proliferation, migration and invasion. As early as 2015, it was pointed out that the high expression of KLF4 was related to the poor overall survival rate and worse metastasis‐free survival rate of OS patients, which seemed to imply that KLF4 promoted OS.^218^ In 2022, the study by Wang et al. further proved that the reduction in ubiquitination of KLF4 made it stably exist in human OS cells and contribute to OS promotion. KLF4 also directly combines with the promoter of its downstream gene, laminin subunit alpha 4 (LAMA4), to boost its transcription, thus suppressing apoptosis of OS cells and facilitating tumour occurrence and migration.^219^ Another study from that year also confirmed that KLF4 expression rose in sarcomas. They discovered that KLF4 was up‐regulated in the process of lncRNA SOX21‐AS1 enhancing the proliferation of human OS cells, and ginsenoside Rg3 decreased OS cell proliferation by targeting down‐regulation of KLF4.^220^ The aforementioned findings show that KLF4 contributes to OS. However, Wang et al. uncovered that KLF4, the direct target of miR‐10b, was down‐regulated to take part in the process of miR‐10b increasing OS cell invasion, which is different from the above research.^221^ This appears to mean that KLF4 inhibits OS in some way. In fact, existing studies show that KLF4 has different effects in different tissues or cancers. For example, KLF4 can reduce the tumorigenicity of gastric cancer and inhibit the formation of lung tumours.^222^, ^223^, ^224^ However, in melanoma, breast cancer and glioma, KLF4 has been proven to inhibit cell apoptosis and promote cell proliferation and metastasis.^219^, ^225^, ^226^, ^227^ The different functions of KLF4 may be related to the huge and complex gene regulatory network of ncRNAs. NcRNAs regulate the expression of many proto‐oncogenes and tumour suppressor genes, and it is still unclear whether other undiscovered potential molecules interfere with the function of KLF4. Meanwhile, some key oncogenes and tumour suppressor factors often mutate, such as GATA3, Kras or p53,^228^, ^229^, ^230^ which may also determine the transformation of the role of KLF4 in ailments. To sum up, the mechanism of KLF4 needs further study.

#### 3.4.2 KLFs and OS cancer stem cells

KLFs can influence cancer stem cells (CSCs)‐like phenotype, OS spheroid formation and chemoresistance. CSCs are a subtype of tumour cells that play a key role in cancer progression, including tumour proliferation, cancer cell metastasis, chemotherapy resistance, spheroidization, recurrence and tumorigenicity.^231^, ^232^, ^233^ CSCs with self‐renewal ability contribute to tumour initiation and treatment resistance, and the existence of OS cancer stem cells (OSCs) is the cause of treatment failure.^223^, ^224^ Recently published work has proved that some KLFs are intimately connected to CSCs‐like phenotypes in OS, primarily including KLF4, KLF8 and KLF11.

KLF4 and KLF8 promote CSCs‐like characteristics of OS. KLF4 has a role in chemoresistance in addition to promoting the formation of OS sarcoma spheres and the cancer stemness of OS cells.^234^ Qi et al. confirmed that the expression of KLF4 was favourably linked with the tumorigenicity of human OS cells and KLF4 improved the ability of OS cells to form spheroids through the p38 MAPK signalling route. Overexpression of KLF4 can up‐regulate the expression of stem cell‐related marker genes (CD133, ALDH1A1 and ABCG2) and activate several signal pathways with self‐renewal ability, including Wnt, Notch, TGF‐β and Hedgehog pathways. Besides, KLF4 has great chemoresistance to adriamycin (ADR) and cisplatin (CDDP), and resists apoptosis induced by ADR or CDDP in OS cells.^235^ At the same time, it has been reported that a chromatin‐binding nucleoprotein high‐mobility group box 1 (HMGB1) can increase the drug resistance by binding with the autophagy regulator Beclin1 (Becn1), while KLF4 at least partially exerts the role of OS chemotherapy resistance by binding and activating the HMGB1 promoter.^236^ It is worth noting that statins can significantly reverse the characteristics and metastasis of OSCs induced by ADR by downgrading KLF4.^237^ This indicates that using statins to inhibit KLF4 seems to be beneficial to the treatment of OS. Consistent with KLF4, KLF8 is also involved in chemoresistance.^238^, ^239^ KLF8 knockout lowers the capacity for tumour formation in vivo and prevents the formation of dense OS sarcoma spheres.^215^ Mechanically, KLF8 inhibits the transcription of miR‐429 by binding to its promoter region, thus directly activating the sex‐determining region Y‐box 2 (SOX2) which can promote tumour cell stemness, and finally promote the CD133‐positive CSCs‐like phenotype.^215^ The data mentioned above imply that KLF4 and KLF8 are crucial players in the development of OS and may be crucial targets in OS treatment.

Inversely, Wang et al. found that KLF11 was down‐regulated in OS patients and identified KLF11 as an inhibitor of inducing OSCs by genome‐wide CRISPR‐Cas9 screening.^240^ The deletion of KLF11 results in increased CSCs‐like properties, particularly in the fraction of CD133 cells, stem cell gene expression, capacity for self‐renewal, tumorigenicity and metastasis, and tolerance to chemotherapy.^240^ Specifically, KLF11 suppresses the transcription activity of yes‐associated protein (YAP)/TEA domain family member 1 (TEAD) by binding to SIN3 transcription regulator family member A (SIN3A)/histone deacetylase (HDAC), which in turn inhibits OSCs. Conversely, YAP/TEAD can encourage KLF11 transcription. However, the methylation of the KLF11 promoter silenced it in OSCs and weakened its inhibition on YAP/TEAD, at which time YAP was continuously activated, and ultimately encouraged the OSCs‐like traits.^240^ The study also demonstrates thiazolidinediones (TZDs), a PPARγ agonist that stimulates KLF11, may become an effective drug to block OSCs. Therefore, a viable avenue for creating more potent OS therapy is to target CSCs through KLFs.

## 4 CONCLUSION AND PERSPECTIVE

**FIGURE 3:**
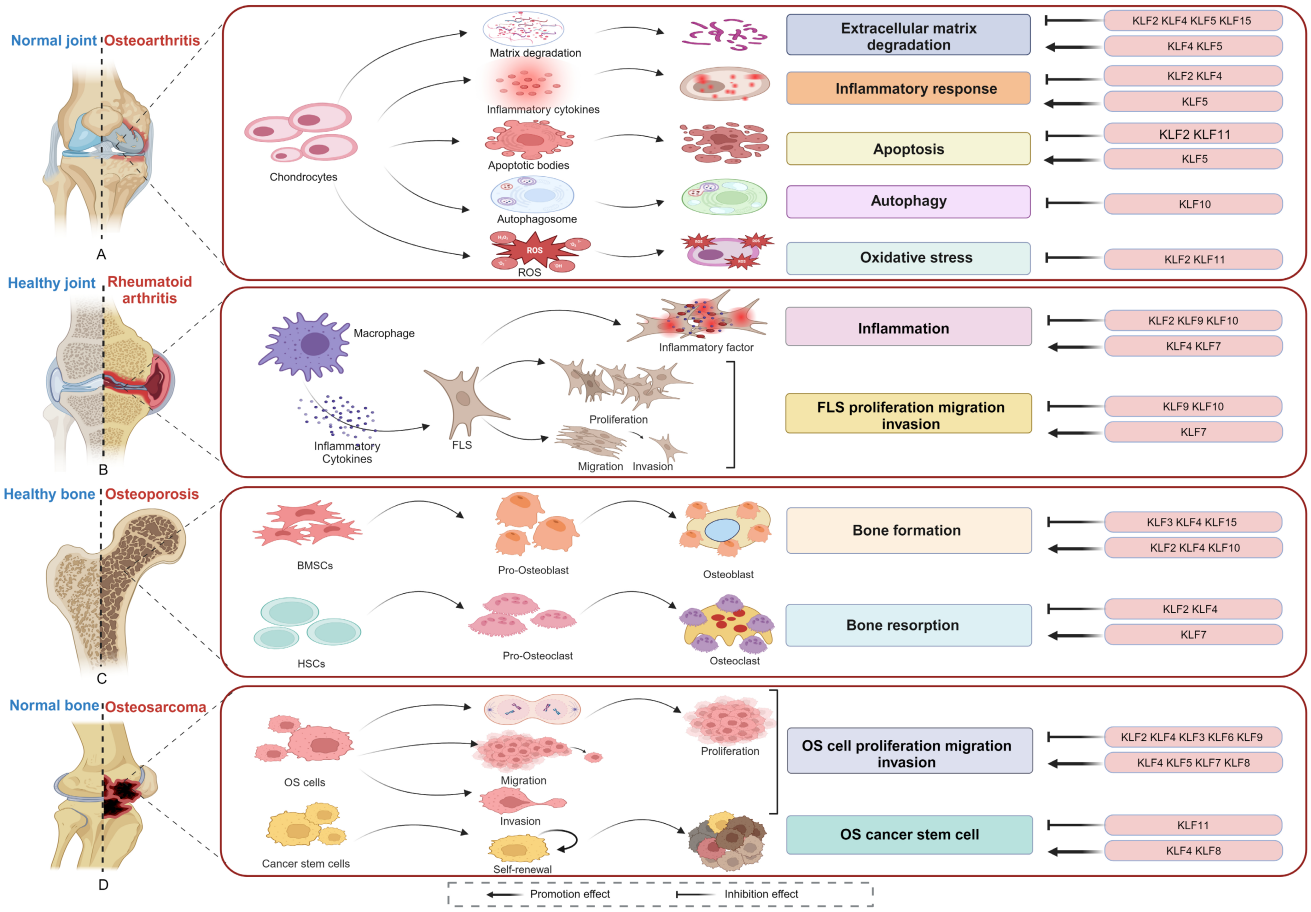
KLFs regulate the pathological process of four kinds of bone diseases.

The most common cause of disability worldwide is bone disease, an inflammatory and degenerative condition that affects muscles, bones and joints. There are currently no viable medications or techniques of treatment for bone illnesses because of the complexity and diversity of their pathophysiology. The data that is now available indicates that numerous members of the KLF family are improperly expressed in bone disorders and take part in the pathophysiological process of bones, making them a very intriguing prospective target for disease therapy. The precise regulatory role of KLFs in bone disorders is still unclear, though. The mechanism by which KLFs drive or inhibit OA, OP, OS and RA is summarized in this review (Figure 3). In OA, KLFs are differentially expressed in articular cartilage, which mainly affects cartilage homeostasis in OA through ECM degradation, inflammatory reaction, chondrocyte apoptosis, autophagy and oxidative stress. Not only can a portion of KLFs be employed as a miRNA target to control OA, but also it may also be controlled by a lncRNA to take part in an inflammatory response. KLFs play a critical role in maintaining bone metabolic equilibrium and bone regeneration in OP by influencing osteogenesis and osteoclast indicators, which in turn impacts bone formation and bone resorption. As the target of ncRNAs in OS, KLFs can not only directly or indirectly control the growth, migration, invasion, metastasis, tumorigenesis and tumour progression of OS cells, but also directly control miRNA to influence OS development. Some KLFs also have a role in OSC expression regulation and chemotherapeutic resistance. In RA, KLFs modulate macrophage polarization, joint tissue deterioration and RA‐FLS proliferation, migration, apoptosis and inflammation. Although it is clear that the KLF transcription factor family is important in bone disorders, the regulation of several KLF family members remains debatable. Therefore, highlighting KLFs' unique function in bone illnesses and elucidating their distinct molecular mechanism in various physiological and pathological phases of bone diseases may help to resolve this issue.

A new concept has been emerging over the past few years, and it is fascinating to see the rapid growth and recent advances in the study of KLFs as a novel and promising area of research. With more in‐depth studies, more KLF family members will be identified as potentially promising biomarkers for monitoring skeletal diseases. The pharmacological targeting of KLFs is an interesting area well worth exploring and could provide new solutions for bone disease management. However, most of the current experimental designs are restricted to cellular and animal experiments, and drug‐targeted KLFs are rarely used in clinical patients. Therefore, accurate and efficient medicine delivery is a challenge that cannot be ignored. Despite the progress in our understanding of KLFs, there are still many unknown issues. To fully understand the properties of KLFs under different physiological conditions, it is of utmost importance to dig deeper into their unidentified structural domains, the target genes they regulate, and the cofactors they interact with. Identifying the signalling pathways that they are involved in is equally essential. In conclusion, these KLF research findings will have an enormous impact on human health and the development of disease.

## AUTHOR CONTRIBUTIONS

**Haixia Wang:** Writing – original draft (equal); writing – review and editing (equal). **Juanjuan Han:** Visualization (equal); writing – review and editing (equal). **Gorbachev Dmitrii:** Conceptualization (equal); supervision (equal). **Ke Ning:** Conceptualization (equal); visualization (equal). **Xin‐an zhang:** Conceptualization (equal); supervision (equal); visualization (equal).

## CONFLICT OF INTEREST STATEMENT

The authors declare no conflicts of interest.

## Data Availability

The data that support the finding of this study are available from the corresponding author upon reasonable request.
